# Physicochemical, microbial, and microbiome dynamics in winery waste composting

**DOI:** 10.1007/s11356-025-36687-8

**Published:** 2025-07-08

**Authors:** Gregoria Mitropoulou, Ioanna Karapantzou, Ioanna Prapa, Dimitra Papanikolaou, Vasileios Charovas, Yiannis Kourkoutas

**Affiliations:** 1 Laboratory of Applied Microbiology and Biotechnology, Department of Molecular Biology and Genetics, Democritus University of Thrace, 68100 Alexandroupolis, Greece; 2 Evritika Kellaria S.A, 68200 Orestiada, Greece

**Keywords:** Composting, Winery waste, Biofertilizer, Microbiome dynamics

## Abstract

Compared to more extensively studied composting substrates like food waste or animal manure, winery waste presents unique challenges and opportunities. Its high content of lignin, cellulose, and polyphenolic compounds demands specific microbial consortia for efficient degradation and can potentially inhibit microbial activity if not properly balanced. In the present study, analysis of winery waste composting that combines traditional microbial enumeration with high-resolution microbiome profiling, an approach rarely applied to this type of agro-industrial residue, was implemented. Moreover, a practical proof-of-concept study, for using the composted product as a partial substrate replacement in grapevine cultivation, closing the loop in vineyard waste management, was conducted. Key parameters, such as moisture content, pH, temperature, conductivity, and C/N ratio were monitored, over a 60-day period, along with changes in enzymatic activity and shifts in microbial populations, indicating dynamic microbial activity. At the end of the process, a reduction in the carbon-to-nitrogen (C/N) ratio was observed, pH was stabilized to neutral values, and dehydrogenases activity was notably decreased. Microbiome analysis revealed eight bacterial and six fungal phyla. *Acidobacteria*,* Armatimonadetes*,* Bacteroidetes*,* Candidatus Saccharibacteria*,* Chloroflexi*,* Cyanobacteria, Planctomycetes* were identified. The *Ascomycota*, *Basidiomycota*, *Chytridiomycota*, *Entomophthoromycota*, *Glomeromycota*, and *Mucoromycota* fungal phyla were also detected. The compost exhibited no phytotoxicity and supported grapevine growth comparable to commercial substrates. Winery waste microbial composting led to stable biofertilizer production, evidenced by physicochemical stability, lack of phytotoxicity, and effectiveness in promoting grapevine growth suggesting the potential of composting as a sustainable waste management solution in the winemaking industry.

## Introduction

Annually, the winemaking industry generates 0.3–0.5 kg of wine by-products per liter, including winter pruning by-products that can be toxic if disposed of without pretreatment, due to high content of organic load and phytotoxic compounds along with high acidity (Karapantzou et al. 2023). These by-products are rich in lignocellulosic material and polyphenols, making them difficult to degrade and potentially phytotoxic if untreated (Niculescu and Ionete 2023). Until recently, such wastes have been generally used for distillation, landfilling, incineration, and/or land-spreading (Karapantzou et al. 2023; Nanni et al. 2021). The last decades, composting has attracted considerable attention as a sustainable and environmentally friendly alternative for the treatment of agro-industrial wastes.

Composting is the natural process of transforming organic matter to fertilizers, rich in essential nutrients for plant growth by microorganisms under controlled conditions (Onwosi et al. 2017). During the bioprocess, organic matter is decomposed with the help of various microbes. The microbial composting process is divided into three phases, based on the temperature of the biomass: (1) the mesophilic phase (20–40 °C), which typically lasts a few days (5–10 days), where bacteria, such as *Bacillus*, *Pseudomonas*, and *Lactobacillus*, dominate and degrade simple sugars and amino acids; (2) the thermophilic phase (45–65 °C), which can last from a few days to several months, dominated by *Thermus*, *Actinobacteria*, and thermophilic fungi like *Aspergillus* and *Penicillium*, which break down complex polymers like cellulose and lignin; and (3) the ripening phase where temperature declines and diverse mesophilic microbes, such as *Streptomyces*, *Trichoderma*, and *Basidiomycota* fungi stabilize the compost and promote humification (Ding et al. 2022; Meena et al. 2021; Rastogi et al. 2020). These microbial transitions are mirrored by shifts in physicochemical parameters, such as moisture, temperature, pH, electrical conductivity (EC), and carbon-to-nitrogen (C/N) ratio. A decreasing C/N ratio, stabilization of pH close to neutral, and reduced oxygen uptake are key indicators of compost maturity and quality (Barros et al. 2021; Lim et al. 2016). Additionally, the high temperatures that develop are the result of intense microbial activity during the first two phases and are responsible for eliminating the pathogenic microorganisms. During the different temperature phases, diverse microbial species dominate. In the first stage, soluble and easily degradable compounds, such as sugars and proteins, are broken down and a sharp rise in temperature is observed. In the second stage, once the temperature exceeds 45 °C, mesophilic microorganisms are replaced by thermophilic microorganisms (Sánchez et al. 2017). In the thermophilic phase, high temperatures accelerate the breakdown of proteins, fats, and complex carbohydrates like cellulose, which is the main structural molecule in plants, and the deactivation of seeds. As the concentration of these compounds is depleted, the temperature gradually decreases and the mesophilic microorganisms take over the final phase (ripening phase). Usually, in this phase, the fungal population increases, while the bacterial population decreases. Aerobic microorganisms prevail when oxygen levels are greater than 5% and their growth is sought, as they are the most important initiators of the process (Meena et al. 2021; Sánchez et al. 2017). The composting process induces substantial shifts in microbial community composition. Initial stages are dominated by fast-growing mesophilic bacteria (e.g., *Proteobacteria*), while later stages see a rise in specialized decomposers and plant-beneficial genera, such as *Bacteroidetes*, *Acidobacteria*, and *Sphingomonadaceae* (Sharma et al. 2022; Asaf et al. 2020). Fungal succession typically progresses from *Ascomycota*-dominated early communities to an increase in *Basidiomycota* and other decomposers involved in lignocellulose breakdown and nutrient recycling (Ding et al. 2022). These microbial transitions are not only vital for compost transformation, but also contribute to the enrichment of the final product with plant growth-promoting organisms. Despite growing interest in winery waste composting, limited studies have examined the microbial ecology and microbiome dynamics throughout the composting process, particularly under semi-pilot-scale, aerated windrow systems using real winery residues.

Compared to more extensively studied substrates like food waste or animal manure, winery waste presents unique challenges and opportunities. Its high content of lignin, cellulose, and polyphenolic compounds demands specific microbial consortia for efficient degradation and can potentially inhibit microbial activity if not properly balanced (Niculescu and Ionete 2023).

The application of winery compost in vineyards offers several potential benefits, including improved soil structure, enhanced nutrient availability, increased microbial biodiversity, and reduced reliance on synthetic fertilizers (Paradelo et al. 2012). However, evaluating compost phytotoxicity and its direct effects on grapevine growth remains essential to ensure its safe and beneficial use in viticulture. Microbial populations during composting are strongly influenced by the type of organic substrate utilized. The composting process, in turn, impacts microbial population changes, reflecting dynamic changes in enzymatic activity, organic matter decomposition, and nitrogen transformations. Nevertheless, the microbiome associations throughout the process remain largely unexplored. Therefore, the aim of the present study was to investigate the microbial composting of winery wastes, focusing on the evolution of physicochemical properties, microbial population dynamics, and microbiome composition over time, and to assess the final compost’s safety and efficacy as a biofertilizer in grapevine cultivation. In this vein, a windrow pile system aerated by agitation was employed, and the germination index test was conducted using barley seeds for assessing the potential phytotoxicity of the final product. Notably, the effectiveness of the final product was further verified in a semi-pilot-scale vineyard cultivation study (proof-of-concept study).

The novelty of this work lies in the integrated, semi-pilot-scale analysis of winery waste composting that combines traditional microbial enumeration with high-resolution microbiome profiling, an approach rarely applied to this type of agro-industrial residue. Moreover, the study provides a practical proof of concept for using the composted product as a partial substrate replacement in grapevine cultivation, closing the loop in vineyard waste management.

From an economic perspective, valorizing winery by-products through composting not only reduces disposal costs and environmental burdens, but also offers a low-cost, circular alternative to commercial substrates and fertilizers, supporting more sustainable and cost-effective viticulture practices.

## Material and methods

### Raw materials

The raw materials used for composting were typical by-products of vinification, including vine shoots, grape stalks and grape pomace. These components are known to be rich in organic matter and polyphenols, and they typically exhibit low pH and high moisture content (Niculescu and Ionete 2023). To initiate the composting process with an optimal C/N ratio of approximately 27.5, as per recommended by Barros et al. (2021), the raw materials were proportionally blended with nitrogen-rich green waste (grass clippings) and soil.

### Windrow pile system and sample collection

**Fig. 1:**
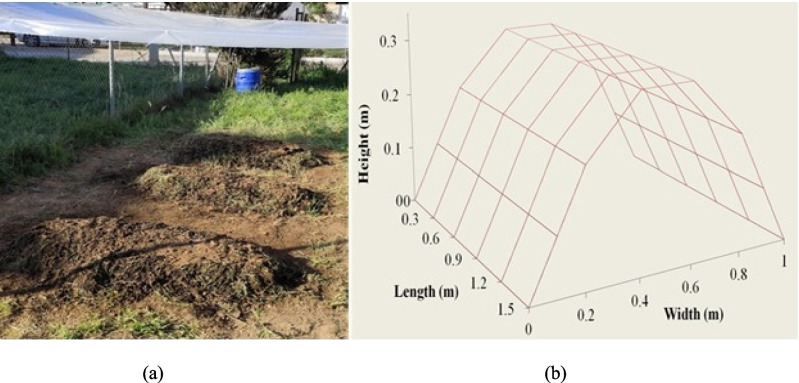
Microbial composting of winery wastes with (**a**) windrow piles and (**b**) dimensional diagram of each pile

The experiment was performed in the field under a canopy, in order to protect the piles from rain. Specifically, three windrow piles with a 3:1 width/height ratio (~ 1 m/0.33 m) (Fig. 1) were loaded with winery waste (plant biomass, vine shoots, grape stalks, and grape pomace from vineyards) and mechanical agitation was carried out every 3 days with an appropriate compost stirrer. The windrow piles were loaded with 70% carbon-rich materials (5-kg vine stems and branches and 15-kg grape marc), 30% nitrogen-rich materials (6-kg grass), and 1.5 kg of soil, according to Barros et al. (2021). Homogeneity and appropriate particle size were achieved with an electrical shredder and sieves. Branches and grass were shredded three times (STIHL, GHE 355) and then passed through sieves with mesh sizes 45 mm, before loading to the windrow piles.

Composite samples (~ 120 g) were collected every 10 and for up to 60 days by mixing five subsamples from different locations of each pile and stored at − 20 ℃ until analysis (Cayuela et al. 2009).

### Chemical analysis

#### Moisture content, pH, conductivity, and temperature

The samples were dried at 121 °C for 12 h, and the moisture content was assessed by measuring the weight loss. For pH determination, the samples were diluted with water at a ratio of 1:10 (weight/volume), and the pH of the resulting supernatant was measured using a pH meter (HANNA, Limassol, Cyprus). Conductivity was monitored with a portable conductivity meter (CON150EUTECH, Thermo Scientific, Singapore). Temperature was determined with a stainless-steel composting waterproof thermometer (temperature range 0–250 °C). 

#### Total carbon (C) and nitrogen (N), C/N ratio, and O_2_ uptake rate

Total carbon (C) and total nitrogen (N) were determined following the methods described before by Yeomans and Bremner (1988) and Bremner (1960), respectively (the minimum detection limit for C was 3% and for N 0.75%). Micro-Kjeldahl distillation method was used to determine NH4 ^+^ -N and NO_3_ ^−^ -N, as described by Raj and Antil (2011). The oxygen (O₂) uptake rate was directly determined using a portable oximeter (linked to a 1.5-m catheter fitted with an O_2_-measuring sensor, HANNA, Limassol, Cyprus).

#### Macro- and micronutrients

The carbonate content was analyzed using a calcimeter (HANNA, Cyprus). The concentrations of phosphorus (P), potassium (K), magnesium (Mg), manganese (Mn), zinc (Zn), iron (Fe), and copper (Cu) levels were quantified via atomic absorption spectrophotometry using a general electric photometer (Agrosoil hardware, Bedfordshire, UK), following the protocol described in EN ISO 9001:2000 (Pinter et al. 2019). The minimum detectable limit was 0.05 ppm.

#### Enzymatic activity

Dehydrogenases activity was determined using a colorimetric method, as previously described (Karapantzou et al. 2023; Schinner 1996). Briefly, compost samples were mixed with CaCO_3_ and 2,3,5-triphenyl-tetrazolium chloride (TTC) solution and incubated at 37 ℃ for 24 h. Following incubation, 25 mL of methanol was added, and the solution was filtered and further diluted in 50 mL of methanol. Absorbance was determined at 485 nm using a microplate reader, and the dehydrogenases activity was calculated according to Karapantzou et al. (2023).

## Microbiological analysis

Microbial populations were analyzed following the protocol described by Karapantzou et al. (2023). Briefly, samples (25 g) were homogenized with sterile 1/4 Ringer’s solution (225 mL), followed by serial dilutions, and total aerobic count (TAC) and levels of *Lactobacillus* spp. (lactic acid bacteria, LAB), *Enterobacteriaceae*, coliforms, *Clostridium* spp., *Escherichia coli*, *Salmonella* spp., as well as yeasts and molds were determined by plate counting. Cell levels were expressed as logcfu/g.

### DNA extraction, PCR amplification, and 16S and ITS rRNA sequencing

To determine microbiome changes during the composting of winery wastes in a windrow pile, Next-Generation Sequencing (NGS) analysis was performed on samples collected at two time points: at the beginning of the composting process (day 0), when raw materials were mixed, and at the end of the process (day 60), when a mature and stable product was obtained. Total DNA extraction was conducted using the NucleoSpin® Soil kit (MACHEREY–NAGEL GmbH & Co., KG, Düren, Germany), according to the manufacturer’s instructions. NGS was carried out by MR DNA using MiSeq sequencing (http://www.mrdnalab.com, Shallowater, TX, USA), as previously described (Karapantzou et al. 2023). In brief, for bacterial analysis, the V1–V3 region of the 16S rRNA gene was amplified with 27 F and 519R primers (5′-AGRGTTTGATCMTGGCTCAG-3′ and 5′GTNTTACNGCGGCKGCTG-3′). Fungal analysis involved amplification of the highly variable internal transcribed spacer (ITS) regions with ITS1 and ITS4 primers (5′-TTGGTCATTTAGAGGAAGTAA-3′ and 5′-TCCTCCGCTTATTGATATGC-3′). Archaeal analysis targeted the 16S rRNA gene region using Arch2A519F and Arch1017R primers (5′-CAGCMGCCGCGGTAA-3′ and 5′-GGCCATGCACCWCCTCTC-3′). PCR amplification consisted of 30–35 cycles, followed by purification of the amplicons using Ampure XP beads. Prepared samples were then processed for Illumina DNA library creation, and sequencing data was analyzed using MR DNA’s proprietary pipeline. Operational taxonomic units (OTUs) were identified by clustering at a 3% divergence threshold, taxonomically classified using BLASTn against curated databases, and low-abundance OTUs (< 0.01) were removed. Raw data analysis and calculation of α-diversity were conducted using the Rhea platform (Lagkouvardos et al. 2017).

### Germination index

The potential phytotoxicity of the final product was evaluated by seed germination and calculation of the germination index, according to Karapantzou et al. (2023) and Paradelo et al. (2012). Briefly, 50 barley seeds were soaked in 50 mL of an aqueous extract (prepared by mixing the compost product with deionized water (1/10 w/v), followed by incubation at 20 ℃ under agitation (60 rpm/min) for 2 h, then filtering with filter paper, and sterilized by a 0.22-µm filter). After 24 h, 15 barley seeds were placed in petri dishes, lined with filter paper soaked with the extract and incubated at 28 ℃ for 5 days. Deionized water was used as the control sample, and the germination index was calculated by the equitation:

Germination index (GI) = 100 × [(*G* × *L*)/(Gc × Lc)], where *G* refers to germination percentage of the compost sample, Gc refers to germination percentage of the control sample, *L* refers to the root size of the compost product, and Lc refers to radicle size of the control sample.

### Proof-of-concept study

The compost product was blended with a commercial substrate (soil) at 25:75 or 50:50 (product:commercial substrate) percentage ratios and used to plant vines in pots. Pots containing 100% commercial substrate were used as control. Each treatment included 10 Moschato Alexandrias (Muscat of Alexandria) vines, with two 1-year-old stumps per pot. The commercial substrate used was Mikskaar 250 L (Mikskaar, Estonia), composed of a black-blonde peat with a ratio of 30–70%. A total substrate volume of 18 L per pot was used, and planting took place in February 2023. For 35 days, shoot growth was monitored. After the 35th day, all leaves from each stump were collected and dried at 65 ℃ for 72 h, and their exact weight before and after drying was determined. The results were expressed as described by Paradelo et al. (2012).

### Statistical analysis

All experiments were conducted in triplicate using 3 independent compost windrow replicates. For physicochemical analysis (e.g., pH, EC) and microbial enumeration (e.g., total aerobic counts, LAB, coliforms), results are presented as mean ± standard deviation (SD) and analyzed *via *one-way ANOVA followed by Tukey’s post hoc test, using Statistica v.12. (StatSoft GmbH, Hamburg, Germany). For microbiome analyses, triplicate samples were collected from each windrow pile at 2 time points (day 0 and day 60). Alpha-diversity indices (Shannon and Simpson indices) were calculated using the Rhea pipeline in R (Lagkouvardos et al. 2017). Taxonomic differences in relative abundances between time points were assessed using ANOVA followed by the Bonferroni post hoc test with *p* < 0.05 considered significant. All sequencing analyses were conducted in triplicate (*n* = 3 per time point), and statistical significance of microbial shifts was determined using non-parametric tests in R.

## Results and discussion

### Physicochemical analysis

Moisture content plays a vital role for the smooth operation of the system, and it was maintained at 40–60%. Excess water in the organic material can create anaerobic conditions and lead to the production of unpleasant odors. Conversely, a lack of water results in dehydration, halting the biological processes and yielding a biologically unstable product (Liang et al. 2003; Diaz et al. 2002). Measurements were made every 10 days, and in case moisture content under 40% was observed, the pile system was wetted manually with water, until the appropriate humidity was reached.

**Fig. 2:**
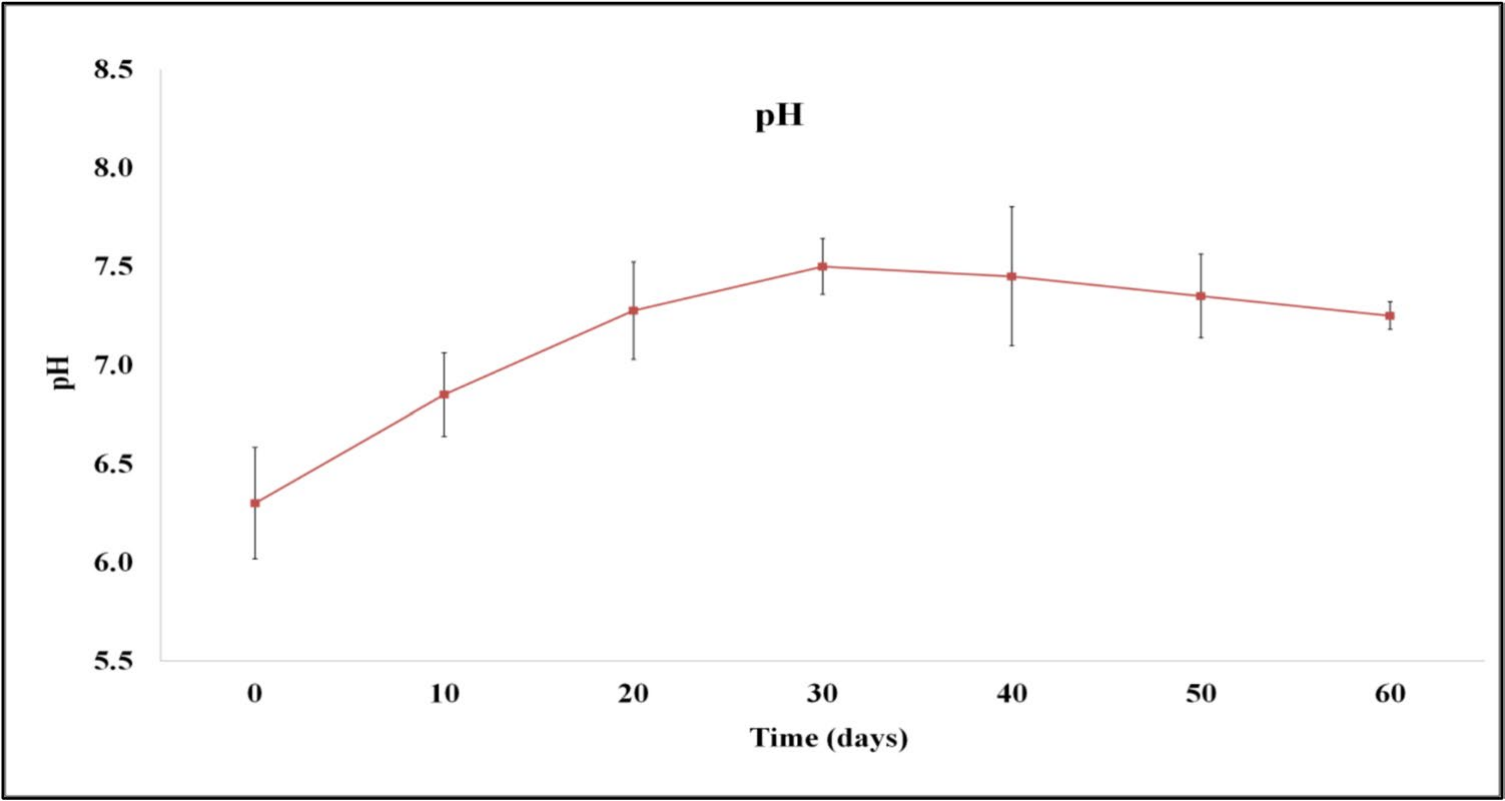
pH changes during microbial composting of winery waste in a semi-pilot-scale windrow pile operated for 60 days

The pH was weakly acidic (6.30) on the first day, while at the end of the process, it varied from weakly acidic to weakly alkaline values (6.88–7.25) (Fig. 2). This change suggested the potential use of the product as a soil conditioner, since crops respond more favorably when the soil pH ranges from a weakly acidic to a weakly alkaline level (Gómez-Brandón et al. 2021).

**Fig. 3:**
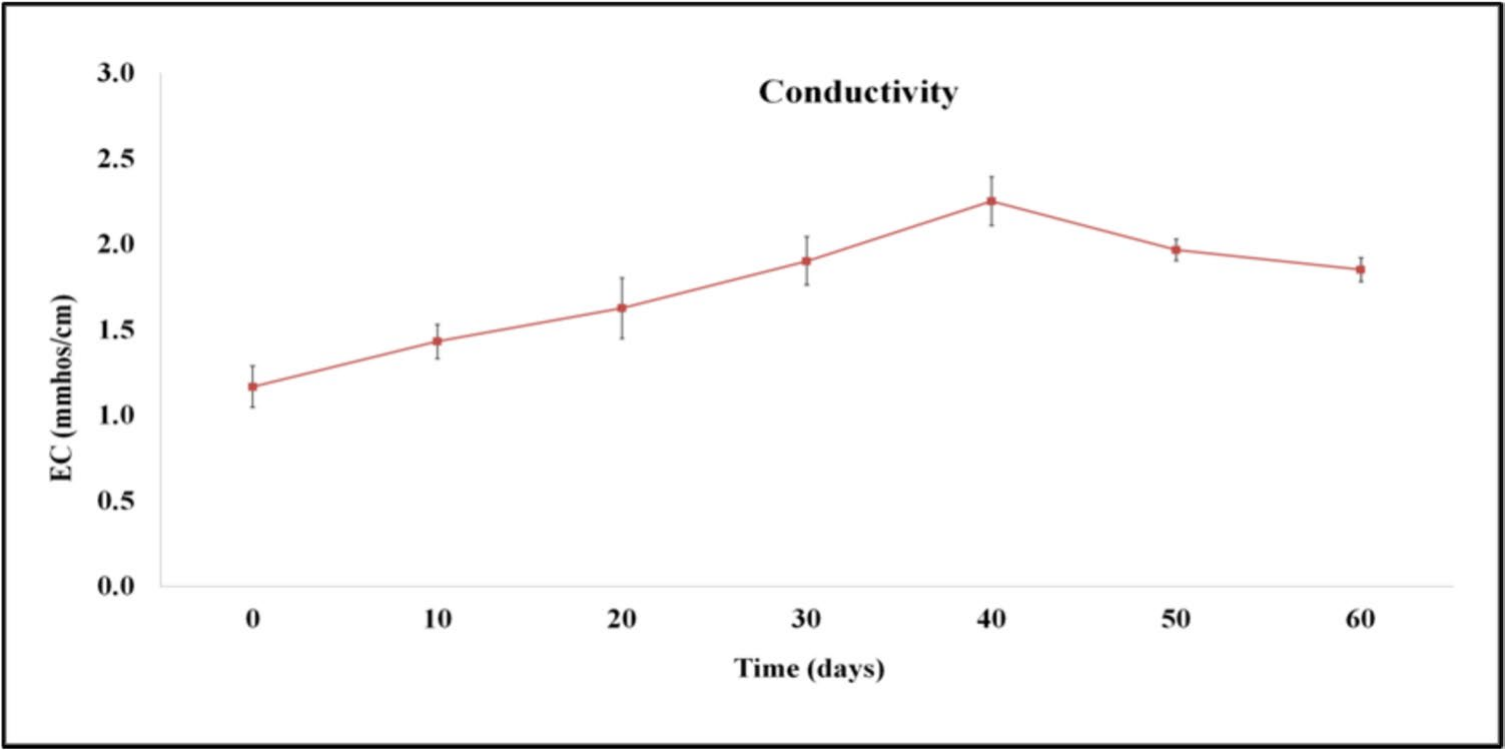
Changes in electrical conductivity expressed as mmhos/cm at 25 ℃ during microbial composting of winery waste in a semi-pilot-scale windrow pile operated for 60 days

Electrical conductivity was significantly (*p* < 0.05) increased from 1.17 to 2.25 mmhos/cm from day 0 to day 40 and then decreased significantly (*p* < 0.05) to 1.85 mmhos/cm at day 60 (Fig. 3), remaining within the upper limit of 3 mmhos/cm proposed for the use of the compost product as a soil conditioner (Soumaré et al. 2002). The concentration of the ions in the composting material along with the type of the material that was used is related to the electrical conductivity. The decrease in electrical conductivity was associated with the stabilization of the product produced (Lim et al. 2016).

**Fig. 4:**
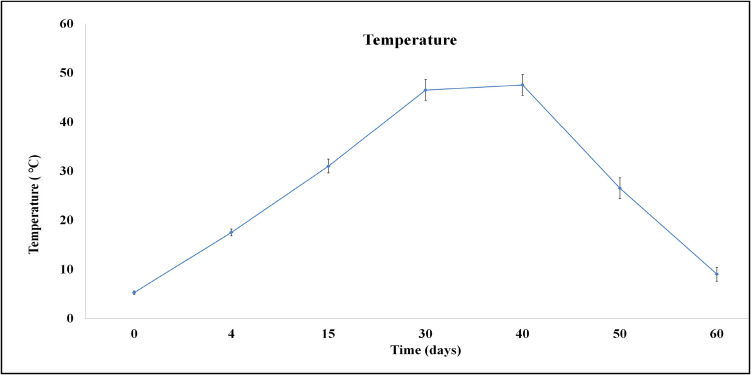
Changes in temperature during microbial composting of winery waste in a semi-pilot-scale windrow pile operated for 60 days

The temperature of the system increased significantly (*p* < 0.05) during the first 15 days (> 30 °C) and ranged between 31.0 and 47.5 ℃ from day 15 to day 40 (Fig. 4). Temperature serves as an indicator of the microbial activity during the process, and levels close to ambient temperature at the end of the process are a good indicator of the end of the bio-oxidative phase (Zhang and Sun 2016). Of note, no fluctuations of the temperature to higher values were noticed.

Total C decreased significantly (*p* < 0.05) from 50.53% of the dry weight (day 0) to 22.61–31.65% (day 60), but no significant (*p* > 0.05) variation in total N between day 0 and day 60 was noted.

**Fig. 5:**
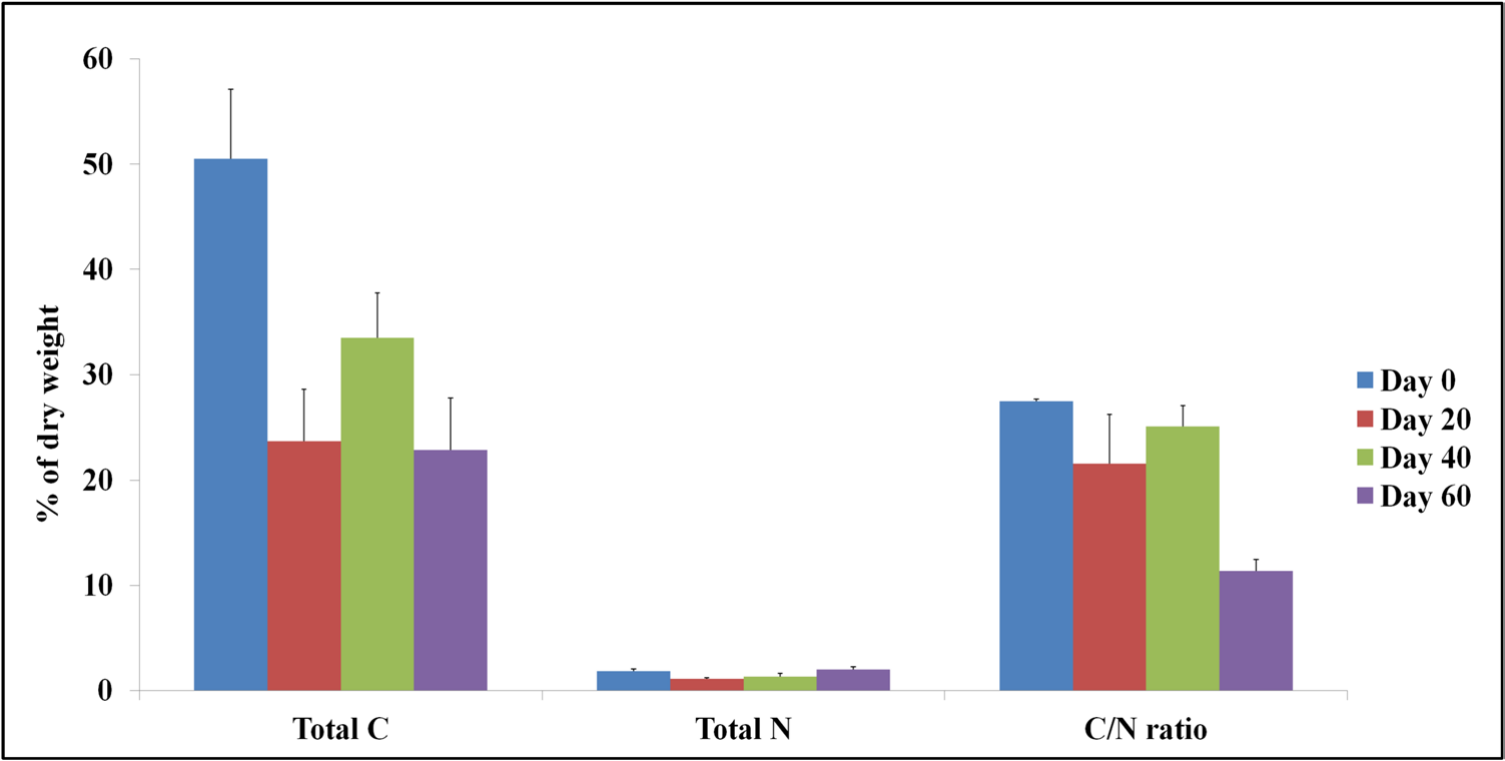
Changes in C/N ratio during microbial composting of winery waste in a semi-pilot-scale windrow pile operated for 60 days

The total carbon (C) content of the composting mixture exhibited a progressive decrease over the course of the process, indicating active organic matter decomposition. This reduction corresponds to the active thermophilic and early maturation phases, during which microbial respiration intensifies, leading to carbon loss mainly in the form of CO_2_. The significant reduction observed during the composting process highlights the efficient mineralization of organic material and supports the ongoing stabilization of the compost. These findings are in line with previous studies that associate carbon loss with microbial activity and compost maturation (Barros et al. 2021; Martínez et al., 2019). Hence, the C/N ratio decreased significantly (*p* < 0.05) from 27.5 on the day of the system initiation to 11.39 on day 60 (Fig. 5). The C/N ratio is used as a factor of stability and maturity of the compost, and its reduction is an important indicator of rapid mineralization and decomposition of the initial raw material (Barros et al. 2021). In this context, the ideal C/N ratio at the beginning of the process is between 25 and 30:1 and tends to be much lower at the end of the process (Barros et al. 2021). Literature reports grape pomace with a C/N ratio ranging between 20 and 50, depending on variety and processing (Pinter et al. 2019). Consistent with the literature (Barros et al. 2021; Mtimkulu et al. 2017), the C/N ratio was significantly reduced at day 60 in our system.

When oxygen is present, certain types of bacteria and archaea successively oxidize NH_4_ ^+^ to NO_3_ ^− ^a bioprocess known as nitrification (Careres et al. 2018). At the initial stages of the composting process, the ammonium (NH_4_ ^+^ -N) concentration is higher and decrease quickly during the thermophilic stage, due to its mineralization to nitrate (NO_3_ ^−^ -N) (Martínez Salgado et al. 2019). Therefore, both NO_3_^−^ concentration and the NH_4_^+^:NO_3_^−^ ratio serve as valuable indicators of compost maturity. According to Sun et al. (2016), nitrogen in nitrate form is more preferable by plants, as it is more easily absorbed; thus, it is essential for the final compost product to have a high nitrate (NO_3_ ^−^ -N) concentration. Ammonium concentration decreased significantly (*p* < 0.05) from day 0 to day 60. Nitrate concentration increased significantly (*p* < 0.05) from day 0 to day 40 and decreased significantly (*p* < 0.05) from day 40 to day 60, but remained significantly (*p* < 0.05) higher than day 0 of the composting process, indicating the maturity of the final compost product. NH_4_ + -N:NO_3_ ^−^ -N ratio decreased significantly (*p* < 0.05) from 1.35 (day 0) to 0.82 (day 60) (Table 1).

**Table 1:** Concentration of NH_4_ ^+^ -N, NO_3_^−^-N, and NH_4_ ^+^ -N:NO_3_^−^-N ratio during composting winery wastes with a windrow pile composting system

Nitrification	Day 0	Day 20	Day 40	Day 60
NH_4_^+^-N (mg/kg)	350 ± 0.12	324.6 ± 0.15	302.5 ± 0.84	298 ± 0.54
NO_3_^−^ (mg/kg)	266.3 ± 1.20	375.7 ± 1.40	399.9 ± 0.42	362.6 ± 0.92
NH_4_^ +^ -N:NO_3_^−^-N	1.35	0.86	0.76	0.82

**Fig. 6:**
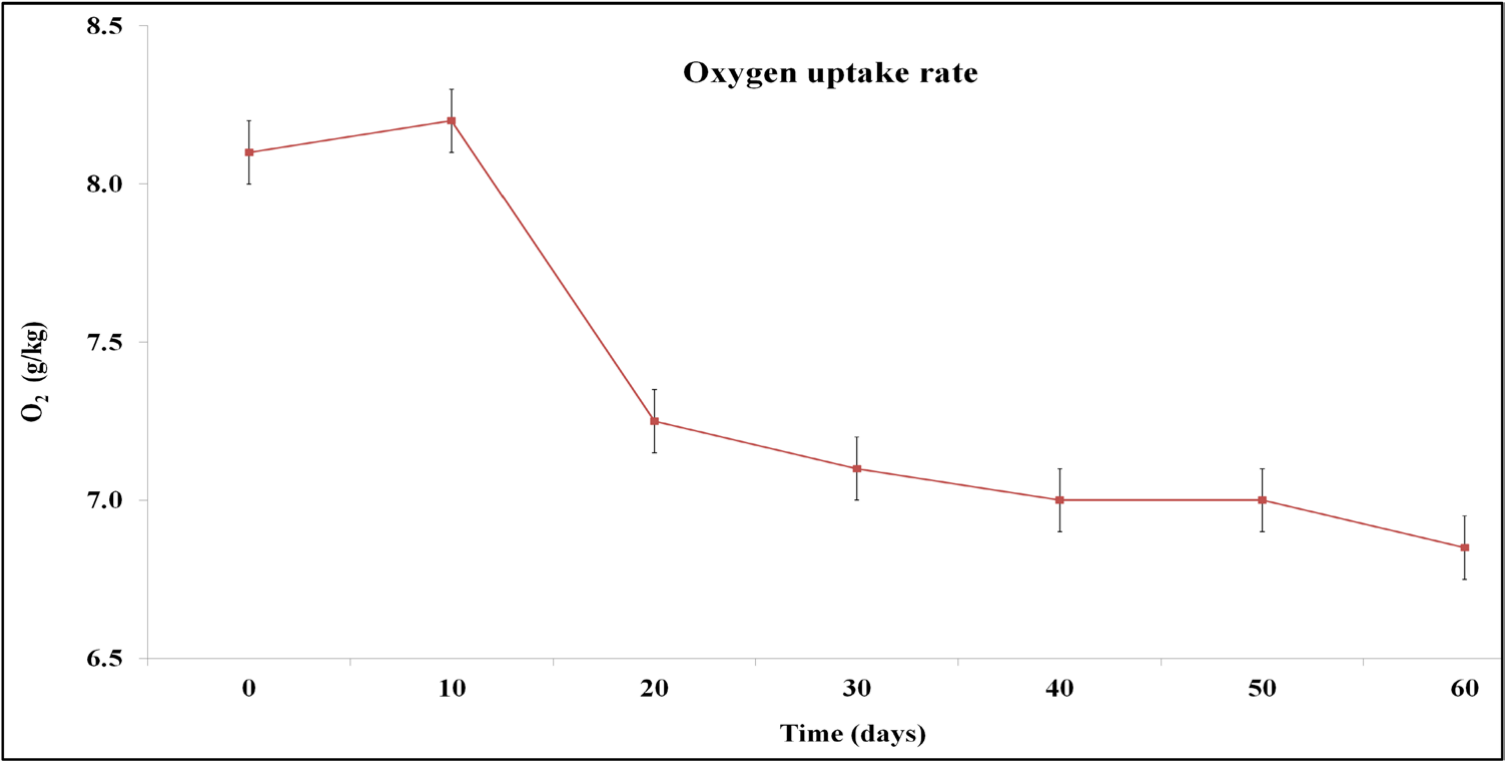
Oxygen uptake rate during microbial composting of winery waste in a semi-pilot-scale windrow pile operated for 60 days, expressed as O_2_ g/kg

Oxygen uptake rate refers to the biological activity of a material, and its reduction at the end of the process is an indicator of the final product stability, as it estimates the readily biodegradable organic matter still present in composting material (Sonowal et al. 2013). The oxygen uptake rate was 8.1 g O_2_/kg at the beginning of the composting process, due to the high availability of biodegradable organic matter. By the end of the process (day 60) this rate significantly decreeased (*p* < 0.05) (6.85 g O_2_/kg), indicating the formation of mature and stable compost (Fig. 6).

### Macro- and micronutrients

The levels of heavy metals and important trace elements of the produced product were determined, and the results are presented in Table 2. In the first 40 days, a significant (*p* < 0.05) increase in the Ca, Mg, K, P, and Fe concentrations was observed, while on day 60, the metal ions levels were significantly (*p* < 0.05) decreased compared to day 40. Many studies have reported both increase and decrease in concentration of the metal ions in the final composting products that were produced from different types of waste (Pinter et al. 2019; Paradelo et al. 2009). Importantly, levels of Mn and Cu ranged within the limits suggested by the European legislation, Regulation (EU) 2019/1009 (https://eur-lex.europa.eu/legal-content/EL/TXT/?uri=CELEX:32019R1009).

**Table 2:** Concentration of important trace elements and metals during composting winery wastes in a semi-pilot-scale windrow pile operated for 60 days

Trace elements/metals	Day 0	Day 20	Day 40	Day 60
Total Ca (% dry weight)	0.10 ± 0.06	1.60 ± 0.60	5.60 ± 0.70	1.50 ± 0.50
Active Ca (% dry weight)	1.40 ± 0.32	0.94 ± 0.18	1.09 ± 0.02	0.13 ± 0.02
CaCO3 (% dry weight)	1.40 ± 0.21	2.50 ± 0.90	6.70 ± 0.70	1.60 ± 0.50
P (ppm)	0.32 ± 0.04	0.26 ± 0.05	0.93 ± 0.25	0.25 ± 0.02
K (ppm)	0.45 ± 0.001	0.36 ± 0.001	4.05 ± 0.14	0.40 ± 0.16
Mg (ppm)	3.92 ± 0.09	1.00 ± 0.27	1.73 ± 0.53	2.00 ± 0.64
Zn (ppm)	580.00 ± 14.10	78.00 ± 39.60	48.90 ± 15.30	83.50 ± 23.30
Mn (ppm)	331.50 ± 2.12	119.25 ± 44.62	40.63 ± 13.61	237.50 ± 24.75
Fe (ppm)	16.50 ± 2.12	44.50 ± 4.95	43.40 ± 4.10	12.00 ± 2.83
Cu (ppm)	32.00 ± 1.41	12.50 ± 0.71	11.50 ± 2.12	33.50 ± 2.12

Our study’s findings align with those of Echeverría-Vega et al. (2024), who also observed significant changes in physicochemical parameters during the composting of winery residues. Both studies reported a decrease in the carbon-to-nitrogen (C/N) ratio and stabilization of pH to neutral values, indicating compost maturity. However, while Echeverría-Vega et al. (2024) utilized sludge additions to adjust moisture content and bulk density, our approach relied on the incorporation of green waste (grass clippings) and soil amendments to achieve optimal composting conditions.

### Enzymatic activity

Maturity of the final product can be also assessed by the determination of enzymatic activity, which is related to the biochemical activity of the compost (Hanc et al. 2022). Specifically, the determination of dehydrogenase activity is associated with the metabolic status of the microbial feedstock (Karwal and Kaushik 2020). Dehydrogenases activity was higher at the beginning of the process and decreased on day 60 (*p* > 0.05) (Table 3). Dehydrogenases play an important role in the biological oxidation of soil organic matter, transferring hydrogen from organic substrates to inorganic receptors (Zhang et al. 2010). The activity of dehydrogenases increases under anaerobic conditions due to the prevalence of anaerobic microorganisms, which are enzyme producers (Wolinska and Stepniewsk 2012). Microbial composting is an aerobic process, and a decline in dehydrogenases activity signifies successful organic matter biodegradation and adequate oxygen availability within the systems being studied.

**Table 3:** Dehydrogenases activity during composting of winery waste in a semi-pilot-scale windrow pile operated for 60 days

	Day 0	Day 20	Day 40	Day 60
Dehydrogenase activity (µg TPF/g dm/24 h)	96.48 ± 18.29	37.41 ± 10.01	41.85 ± 15.64	30.94 ± 4.84

### Microbial populations

**Fig. 7:**
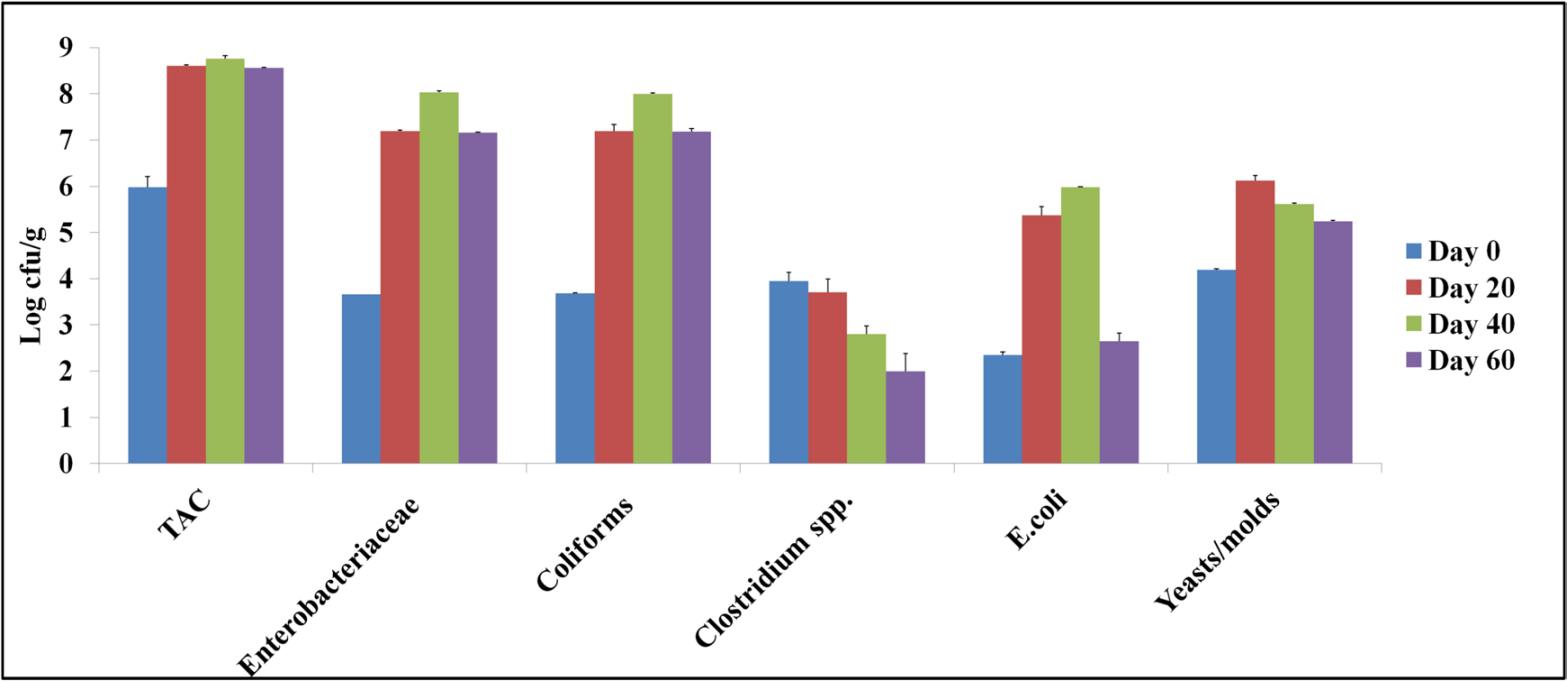
Microbial population levels during microbial composting of winery waste in a semi-pilot-scale windrow pile operated for 60 days, expressed as logcfu/g of compost. TAC: total aerobic counts

Microbial populations were also determined at regular intervals during the process (Fig. 7). A significant increase in the cellular levels of all microbial species, except *Clostridium* spp., was observed on day 20, due to the high amount of biodegradable organic matter available to microbes, and a significant reduction (*p* < 0.05) was noticed on day 60, as mature compost was produced. *Clostridium* spp. levels decreased significantly (*p* < 0.05) during the process. According to the European Regulation (EU) 2019/1009 (europa.eu), *Salmonella* spp. should be undetectable in the final product, while *Escherichia coli* levels should range < 3 logcfu/g. Importantly, the above criteria were met in our case. Subirats et al. (2022) indicated the suitability of the aerobic composting process as a method to reduce the levels of spore forming or pathogenic bacteria in solid wastes, such as *Clostridium* spp. and fecal coliforms, which is confirmed in our study, as the sanitization of the final product was accomplished. Low levels of coliforms and *Escherichia coli* were detected in the early stages of composting and were further significantly reduced (*p* < 0.05) by the end of the process (day 40 to day 60), meeting the safety thresholds set by the European Regulation (EU) 2019/1009. The presence of these organisms is not uncommon in plant-based composting substrates. Coliforms and *E. coli* may originate from several sources, including vineyard soil residues, grape pomace surfaces, wine lees, or cross-contamination from harvesting and transport equipment (Subirats et al. 2022). Soil added to the composting piles as inoculum could also have contributed small initial amounts of environmental coliforms. The observed decline during composting is consistent with effective thermophilic sanitization and supports the microbiological safety of the final product for agricultural application. Of note, TAC,* Enterobacteriaceae*, and yeasts/molds levels ranged between 3.66 and 8.76 logcfu/g.

### Next-Generation Sequencing DNA analysis

Bacteria and fungi play an important role in the decomposition of organic matter. The NGS analysis identified 206 bacterial, 417 fungal, no archaeal OTUs, and revealed substantial shifts in the microbial communities between the beginning and the end of the composting process (*p* < 0.05). Diversity indices not only provide important information about the rarity, but also about the likelihood of certain species being dominant or commonly found (Yanni et al. 2020). In this vein, Shannon and Simpson indices (α-diversity) were used (Karapantzou et al. 2023; Yanni et al. 2020). The Shannon index increased (*p* < 0.05), while the Simpson index decreased significantly (*p* < 0.05) for bacteria, but no significant (*p* > 0.05) changes were observed for fungi (data not shown).

In total, eight bacterial phyla were detected. Specifically, *Acidobacteria*, *Armatimonadetes*, *Bacteroidetes*, *Candidatus* Saccharibacteria, *Chloroflexi*, C*yanobacteria*, *Planctomycetes*, and *Proteobacteria* were the bacterial phyla identified. *Proteobacteria* were the most abundant at the beginning of the process, representing 99.37% of the total identified sequences. At day 60, *Proteobacteria* decreased significantly (*p* < 0.05), while *Acidobacteria*, *Armatimonadetes*, and *Bacteroidetes* increased significantly (*p* < 0.05) (Table 4).

**Table 4:** Changes in bacteria phylum abundances (%) during microbial composting of winery waste (grapes, lees, etc.) in a semi-pilot-scale windrow pile operated for 60 days

Bacterial phyla	Day 0	Day 60
*Acidobacteria*	0.13 ± 0.05	1.98 ± 0.92
*Armatimonadetes*	0.03 ± 0.001	0.28 ± 0.12
*Bacteroidetes*	0.37 ± 0.16	16.62 ± 1.36
*Candidatus Saccharibacteria*	0.01 ± 0.001	0.04 ± 0.00
*Chloroflexi*	0.01 ± 0.001	0.06 ± 0.02
*Cyanobacteria*	0.02 ± 0.001	0.02 ± 0.001
*Planctomycetes*	0.03 ± 0.001	0.03 ± 0.001
*Proteobacteria*	99.37 ± 0.26	80.97 ± 0.32

**Fig. 8:**
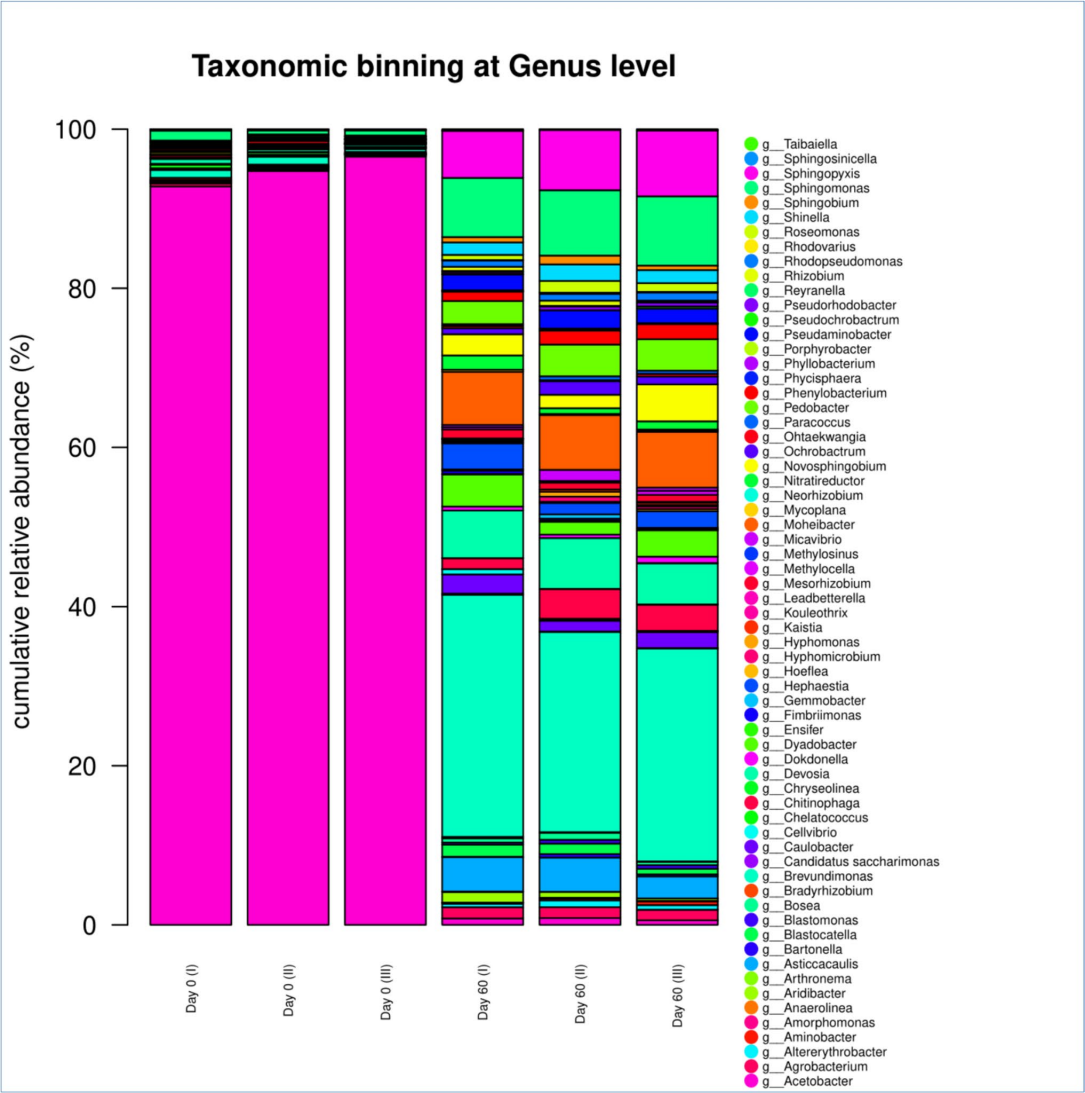
Bar plot of the mean relative genus abundances of the predominant bacteria during microbial composting of winery waste in a semi-pilot-scale windrow pile operated for 60 days. I, II, and III refer to the triplicate samples

In genus level, 65 bacterial genera were detected at the beginning and the end of the bioprocess (Fig. 8). The most abundant genus on day 0 was *Acetobacter* (94.70%), while the remaining genera ranged < 1%. At day 60, the most abundant genera were *Brevundimonas* (27.46%), *Sphingomonas* (8.12%), *Sphingopyxis* (7.24%), *Moheibacter* (6.86%), *Devosia* (5.83%), *Asticcacaulis* (3.79%), *Pedobacter* (3.62%), *Novosphingobium* (3.00%), *Dyadobacter* (2.99%), *Chitinophaga* (2.78%), *Hephaestia* (2.27%), *Pseudaminobacter* (2.05%), *Caulobacter* (1.93%), *Shinella* (1.76%), *Phenylobacterium* (1.62%), *Agrobacterium* (1.37%), *Nitratireductor* (1.18%), *Blastocatella* (1.18%), *Ochrobactrum* (1.16%), and *Roseomonas* (1.08%), while the remaining genera ranged < 1%. At day 60, the bacterial genera abundance of *Agrobacterium*, *Altererythrobacter*, *Aminobacter*, *Asticcacaulis*, *Blastocatella*, *Blastomonas*, *Bosea*, *Brevundimonas*, *Caulobacter*, *Chitinophaga*, *Devosia*, *Dokdonella*, *Dyadobacter*, *Fimbriimonas*, *Hephaestia*, *Hoeflea*, *Hyphomicrobium*, *Kaistia*, *Mesorhizobium*, *Methylocella*, *Moheibacter*, *Mycoplana*, *Nitratireductor*, *Novosphingobium*, *Ochrobactrum*, *Paracoccus*, *Pedobacter*, *Phenylobacterium*, *Phyllobacterium*, *Pseudaminobacter*, *Pseudorhodobacter*, *Rhodopseudomonas*, *Rhodovarius*, *Roseomonas*, *Shinella*, *Sphingobium*, *Sphingomonas*,* Sphingopyxis*, and *Sphingosinicella* increased significantly (*p* < 0.05), while *Acetobacter* and *Methylosinus* decreased significantly (*p* < 0.05). Of note, no significant changes were detected for the rest of the bacterial genera (*p* > 0.05).

The genus *Brevundimonas* has been isolated from a wide range of environmental conditions, and it often promotes plant growth (Sharma et al. 2022). The genera *Sphingopyxis*, *Sphingomonas*, and *Novosphingobium* are well known for their role in environmental nutrient cycling, and they produce beneficial phytohormones that promote plant growth (Verma et al. 2020; Asaf et al. 2020). *Moheibacter* species are known for colonization of soils rich in organic matter (Yu et al. 2022; Liu et al. 2021; Schauss et al. 2016; Zhang et al. 2014) and are commonly found in compost products, suggesting that *Moheibacter* species can decompose and utilize organic material (Yu et al. 2022). *Phenylobacterium* has the ability to degrade complex carbohydrates, such as cellulose and possibly lignin (Milkereit et al. 2021), grape seeds, and grape stalks that have a high content of carbohydrates and lignocellulosic materials (Niculescu and Ionete 2023). *Asticcacaulis*, *Caulobacter*, *Devosia*,* Ochrobactrum*, *Pedobacter*, and *Shinella* genera belong to the group of plant growth promoting bacteria named Rhizobia (Okazaki et al. 2021; Morais et al. 2019; Verma et al. 2018). Their main function is to fix atmospheric nitrogen together with plants and they have been widely studied as symbiotic plant growth promoters (Chhetri et al. 2021; Verma et al. 2018). The diversity in the percentage abundance in bacteria genera reveals the successful biotransformation performed, resulting in a final product rich in plant growth promoting bacteria.

Regarding the fungal phyla, *Ascomycota*, *Basidiomycota*, *Chytridiomycota*, *Entomophthoromycota*, *Glomeromycota*, and *Mucoromycota* were detected. The predominant fungal phylum was *Ascomycota* (97.43%), and its percentage decreased significantly (*p* < 0.05) after 60 days of microbial composting, while *Basidiomycota* OTUs increased significantly (*p* < 0.05) at the end of the process. The changes observed for the rest of the fungal phyla were not significant (Table 5).

**Table 5:** Changes in fungal phylum abundances (%) during microbial composting of winery waste (grapes, lees, etc.) in a semi-pilot-scale windrow pile operated for 60 days

Fungal phyla	Day 0	Day 60
*Ascomycota*	97.43 ± 0.64	90.28 ± 3.05
*Basidiomycota*	2.35 ± 0.64	8.16 ± 2.57
*Chytridiomycota*	0.01 ± 0.001	0.01 ± 0.001
*Entomophthoromycota*	0.01 ± 0.001	0.01 ± 0.00
*Glomeromycota*	0.03 ± 0.01	0.86 ± 0.40
*Mucoromycota*	0.18 ± 0.01	0.68 ± 0.09

**Fig. 9:**
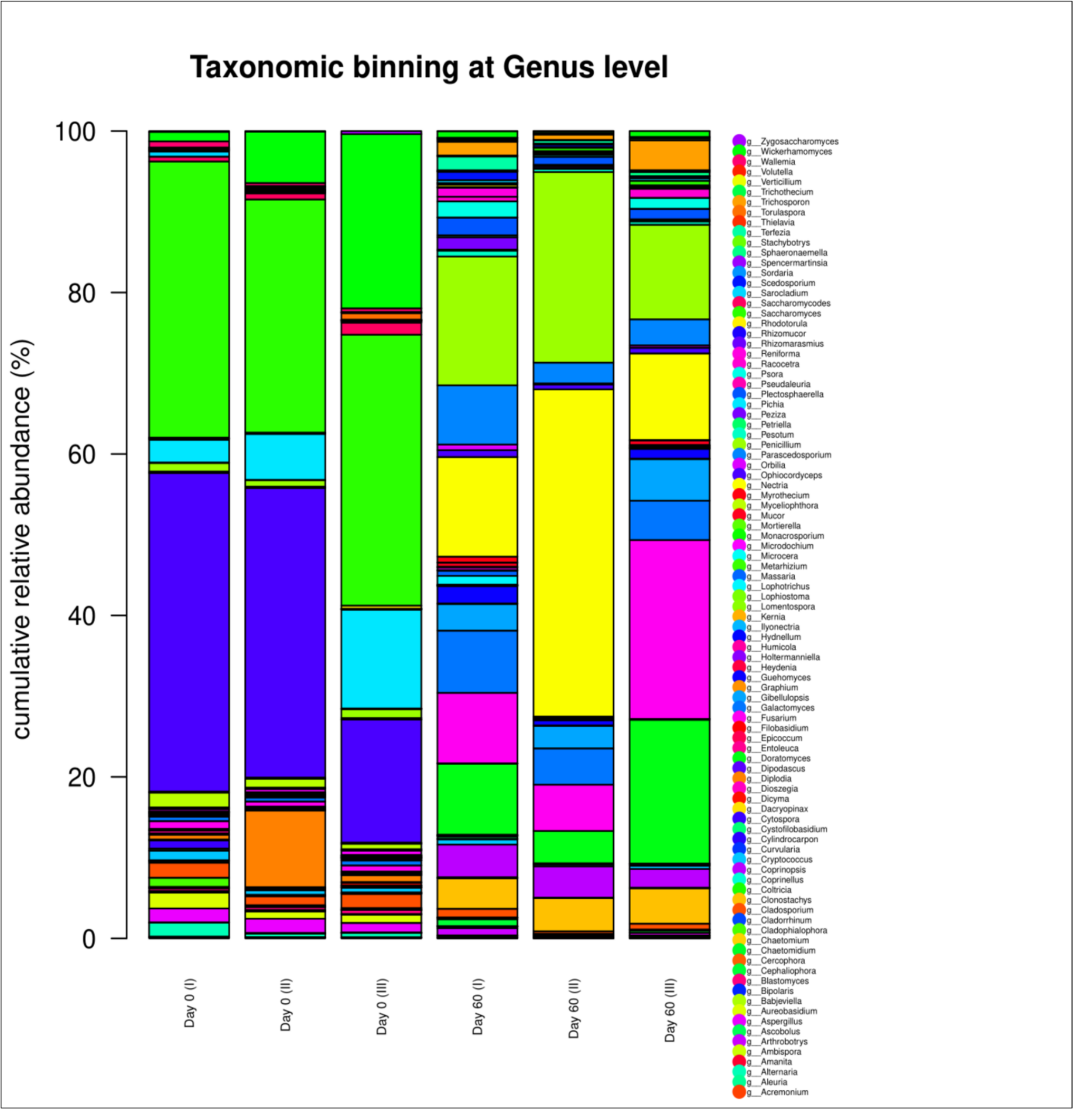
Bar plot of the mean relative genus abundances of the predominant fungi during microbial composting of winery waste in a semi-pilot-scale windrow pile operated for 60 days. I, II, and III refer to the triplicate samples

At genus level, the most abundant fungi genera on day 0 were *Saccharomyces* (32.21%), *Ophiocordyceps* (30.19%), *Wickerhamomyces* (9.70%), *Pichia* (6.92%), *Diplodia* (3.66%), *Cladosporium* (1.57%), *Aspergillus* (1.54%), *Aureobasidium* (1.29%), and *Myceliophthora* (1.20%). At day 60, the most abundant fungi genera were *Nectria* (21.20%), *Penicillium *(17.10%),* Fusarium *(12.21%), *Doratomyces* (10.19%), *Galactomyces* (5.68%), *Parascedosporium* (4.38%)*, Clonostachys* (4.10%), *Gibellulopsis* (3.75%), *Coprinopsis* (3.41%), *Trichosporon* (2.02%), *Plectosphaerella* (1.50%), *Guehomyces* (1.34%), and *Psora* (1.20%) (Fig. 9). The fungal genus abundance of *Amanita*, *Clonostachys*, *Coprinopsis*, *Galactomyces*, *Gibellulopsis*, *Graphium*, *Parascedosporium*, *Penicillium*, *Pesotum*, *Petriella*, *Plectosphaerella*, *Verticillium*, and *Volutella* increased significantly at day 60 (*p* < 0.05), while the fungal genus abundance of *Acremonium*, *Aspergillus*, *Aureobasidium*, *Babjeviella*, *Blastomyces*, *Coltricia*, *Curvularia*, *Dioszegia*, *Epicoccum*, *Filobasidium*, *Hydnellum*, *Mortierella*, *Microdochium*, *Myceliophthora*, *Ophiocordyceps*, *Saccharomyces*, *Saccharomycodes*, *Spencermartinsia*, and *Wallemia* decreased significantly (*p* < 0.05).

*Parascedosporium* is a fungus that produces enzymes active against carbohydrates (carbohydrate-active), which, when grown on a substrate with lignocellulose (stems are rich in lignocellulose), enhances its decomposition (Lackner and Hoog 2011). *Guehomyces* is associated with the activity of laccase, an oxidase of starches, and possibly degrades organic matter (Ding et al. 2022). *Gibellulopsis* has been reported not only in composting products by other studies, but also in rotting root systems, and it is related to the carbohydrate content of the substrate (Tang et al. 2022; Hao et al. 2022). According to the literature, the genus *Galactomyces* has been associated with competitive mechanisms against phytopathogenic fungi and can be used as a biocontrol agent (Cai et al. 2020). The presence of the genus *Penicillium* is considered beneficial, since it promotes the solubilization of phosphorus from organic matter through the action of phosphatases, acting as a biostimulator (Gómez-Guiñán 2014). Some species of the genus *Fusarium* are pathogenic, and thus the phytotoxicity of the final product should be evaluated before field application.

The progression of microbial communities throughout the composting process was closely linked to changes in physicochemical parameters, such as temperature, pH, moisture content, and C/N ratio. In addition to temperature, pH and bulk density played a critical role in shaping microbial community dynamics throughout the composting process. pH values evolved from weakly acidic at the beginning (6.30) to near-neutral by the end of the process (7.25), which likely influenced microbial succession. Furthermore, although bulk density was not directly measured, the compost structure determined by the proportion of vine shoots, pomace, and grass likely affected aeration and moisture retention. These factors are known to influence oxygen availability and microbial colonization patterns (Rastogi et al. 2020). An overly compact matrix may suppress aerobic microbial populations by limiting oxygen diffusion, whereas adequate porosity promotes the growth of thermophilic and cellulolytic microbes during the active composting phases.

Early acidic conditions favored the dominance of *Acetobacter*, while the stabilization to neutral pH values supported a broader microbial diversity, particularly the emergence of *Actinobacteria*, *Sphingomonadaceae*, and *Basidiomycota* fungi. These shifts are consistent with previous studies showing that microbial activity and diversity are enhanced under near-neutral conditions, which optimize enzyme function and nutrient availability (Gómez-Brandón et al. 2021; Ding et al. 2022).

During the initial mesophilic and early thermophilic phases, the availability of readily degradable substrates like sugars and amino acids supported the proliferation of fast-growing bacteria, particularly members of the *Proteobacteria* phylum, including genera like *Acetobacter*. This phase coincided with intense microbial metabolism, reflected in the high oxygen uptake rate and increased dehydrogenases activity. As composting advanced into the thermophilic phase, the elevated temperatures (over 45 °C) favored thermotolerant and thermophilic taxa, while simultaneously suppressing mesophilic microbes and potential pathogens, such as *Clostridium* and *Escherichia coli*. The reduction in the C/N ratio over time indicated progressive mineralization of organic matter, supporting the growth of microbial taxa capable of degrading complex polymers like lignin and cellulose (e.g., *Chitinophaga*, *Fusarium*). Similarly, pH stabilization toward neutral values and a decrease in electrical conductivity suggested a transition toward a more mature and stable compost environment, which likely supported the re-establishment of fungal taxa, such as Basidiomycota, known for their role in humification. These physicochemical trends mirrored shifts in microbial diversity, with an increase in bacterial alpha-diversity and restructuring of fungal communities, highlighting the reciprocal relationship between microbial succession and the biochemical transformation of composting material. The microbial community acted not only as a driver, but also as a sensitive indicator of composting progress and stability.

In terms of microbial dynamics, Echeverría-Vega et al. (2024) also noted an increase in bacterial diversity over time and a reduction in fungal diversity during the thermophilic phase, with partial recovery during maturation, and they highlighted the dominance of thermophilic bacteria and a decline in fungal diversity due to elevated temperatures. Similarly, our results demonstrated a succession of microbial communities, with thermophilic bacteria prevailing during high-temperature phases and a resurgence of fungal populations as temperatures decreased.

### Germination index

Majlessi et al. (2012) indicated that even if the final compost product is stable (low C/N ratio, neutral pH, and low electrical conductivity), it may still contain phytotoxic compounds, and in order to mitigate environmental risks, it is essential to check the potential phytotoxicity of compost products prior to application to soil. The germination index (GI) is an extremely reliable indicator for determining the maturity stage of organic fertilizers, as it reflects whether the compost is non-toxic, safe, and beneficial (Sokač Cvetnić et al. 2024). Notably, GI values greater than 80 indicate the absence of phytotoxicity, suggesting that the compost can be safely used as an organic fertilizer (Paradelo et al. 2012; Michailides et al. 2011). Therefore, the final compost product (day 60) was assessed for potential phytotoxicity using barley seeds, and both germination and radicle growth were measured. The GI was found to be 133.99, indicating the maturity and stability of the final compost product, well above the 80% threshold typically used to define compost maturity and safety (Sokac Cvetnic et al. 2024; Paradelo et al. 2012). This strong result indicated the absence of inhibitory substances, such as organic acids or ammonia, and confirms the compost’s suitability for agricultural use.

#### Proof-of-concept study

The results from GI showed no phytotoxicity, and therefore, the final compost product was used as a substrate in grapevines’ growth in the proof-of-concept study. Specifically, the final product was mixed with a commercial substrate at 25 and 50%. A ratio greater than 50% was not evaluated, as according to Paradelo et al. (2012), 50% is considered the upper limit for using winery waste compost products for use as a plant growth substrate. The effectiveness of the final product was assessed by comparing the percentage yield of grapevine leaf dry matter to that of the control samples. Values 110 ± 1.3 and 90 ± 1.8 for 25:75 and 50:50 (compost product:commercial substrate), respectively, were recorded. Hence, the final product was considered suitable as a substrate in grapevine growth, as percentage yields ≥ 90% were determined. Of note, the 25:75 (product:commercial substrate) ratio resulted in a significantly higher percentage yield of grapevine leaf dry matter compared to the 50:50 ratio.

These results demonstrated that the composted product can effectively replace up to 50% of commercial substrate without compromising plant performance. Interestingly, the 25:75 ratio even resulted in higher dry matter yield than the commercial substrate, possibly due to improved microbial activity and nutrient availability in the blend.

These findings are consistent with Paradelo et al. (2012), who reported similar results when using composted winery residues in pot trials. Our study further contributed by demonstrating the successful implementation of composting under windrow conditions, while also validating both microbiological safety and functional performance of the final product. The combination of high GI, favorable physicochemical properties, and positive vine growth outcomes supported the use of winery waste compost as a cost-effective and environmentally sound amendment for vineyard applications.

#### Technological considerations

The results of our study are not only broadly consistent with prior works, but also demonstrate distinct advantages in terms of composting efficiency and product application. For instance, Ivanović et al. (2024) reported composting durations of up to 60 days using grape marc with various co-substrates, observing gradual reductions in C and organic matter content and moderate biodegradability (15–25%). However, some treatments failed to reach thermophilic conditions, likely due to the inhibitory effects of polyphenols or insufficient porosity. In contrast, our windrow system consistently achieved thermophilic temperatures and rapid C/N ratio reduction, suggesting more efficient microbial degradation. Similarly, Semitela et al. (2019) showed that mesophilic composting of winery waste and grape stalks could produce mature compost under specific aeration and temperature regimes, though no thermophilic stage was observed. While that study emphasized physicochemical and germination outcomes, our approach incorporated both microbial profiling and vineyard application, offering a more holistic approach.

Martínez-Salgado et al. (2019) monitored grape pomace compost over 180 days, using enzymatic, microbial, and phytotoxicity parameters to evaluate maturity. Their results highlighted strong correlations between microbial function and humification, paralleling our own findings on microbial succession and dehydrogenases activity. However, our process reached compost maturity within 60 days, likely due to improved substrate blending, aeration, and pile structure. Finally, Echeverría-Vega et al. (2024) conducted a study over a period of 6 months, whereas our composting process reached maturity within 60 days—likely due to differences in initial material composition, pile management, and environmental conditions. Additionally, our study incorporated a GI assessment and a practical application of the compost in grapevine cultivation, providing insights into the agronomic benefits of the composted product.

## Conclusion

This study demonstrated the feasibility and effectiveness of composting winery waste using a semi-pilot-scale windrow system, offering a sustainable solution for managing agro-industrial by-products. Unlike previous studies that focused primarily on physicochemical parameters or small-scale composting, our work integrated microbial enumeration, high-resolution microbiome profiling, and practical evaluation of the compost as a soil amendment in grapevine cultivation.

Key findings included a substantial reduction in the C/N ratio from 27.5 to 11.4, a microbial shift from mesophilic *Acetobacter*-dominated communities to functionally diverse genera, such as *Brevundimonas* and *Sphingopyxis*, and a high GI (133.99), confirming the absence of phytotoxicity.

These results underscore the environmental and agronomic value of converting winery residues into stable, nutrient-rich compost, supporting circular economy principles and reducing the reliance on synthetic inputs in viticulture. Future research should focus on long-term field validation, functional characterization of microbial communities during compost maturation, and evaluation of compost effects on grape yield and soil health under real vineyard conditions.

## Data Availability

All data are available upon request.
